# Supplementary material for: Improving the Clinical Application of Natural Killer Cells by Modulating Signals Signal from Target Cells
Source: Int J Mol Sci. 2019 Jul 15;20(14):3472. doi: 10.3390/ijms20143472 (PMC6679089; doi:10.3390/ijms20143472)
Supplement: Supplementary file 1 [file ijms-20-03472-s001.pdf]

SUPPLEMENTARY INFORMATION

Supplementary table

CD marker	Fluorophore	Cat.no.	Company
CD45	BV510	563204	BD Bioscience
CD16/56	PE	ED7054	Exbio
CD3	Pacific Blue	A93687	Beckman Coulter
CD56	APC-Cy7	362512	Biolegend
CD25	PE-Cy7	356108	Biolegend
NKp44	APC	325110	Biolegend
CD16	FITC	1F-646-T100	Exbio
NKG2D	PE	12-5878-41	eBioscience
CD158a (KIR2DL1)	PE	130-103-934	Miltenyi
CD158b (KIR2DL2/DL3)	APC	130-092-617	Miltenyi
CD158b2 (KIR2DL3)	FITC	130-100-125	Miltenyi
CD158e (KIR3DL1)	FITC	130-092-568	Miltenyi
CD158e/k (KIR3DL1/DL2)	PE	130-095-205	Miltenyi
CD158f (KIR 2DL5)	APC	130-098-569	Miltenyi
CD45	Krome Orange	B36294	Beckman Coulter
CD16	PerCP	PC-646-T100	Exbio
HLA-A,B,C	PE	311406	Biolegend
7-AAD	NA	EXB0026	Exbio

Supplementary table S1: An overview of the antibodies used in the study.

Supplementary figures

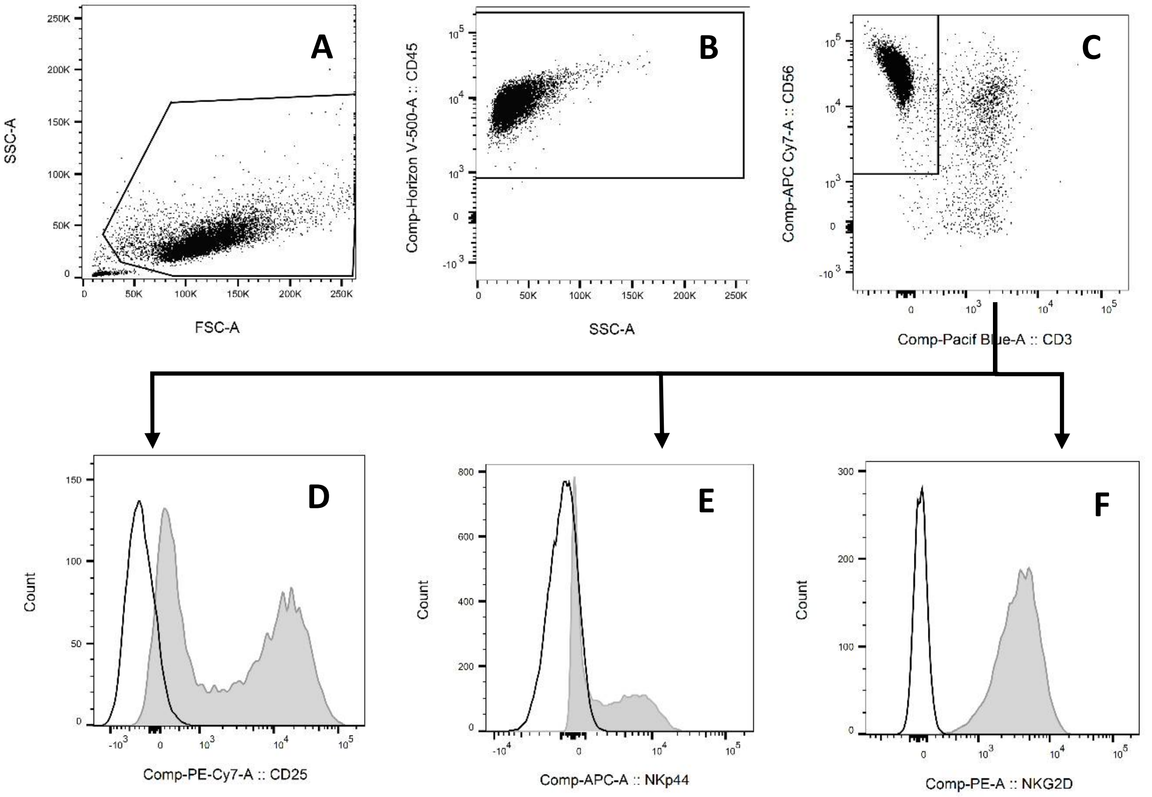

Supplementary figure S1. NK cells were gated using followed gating strategy. First, debris was excluded in FSC/SSC dot-plot (A), then CD45 positive leukocytes were selected (B). NK cells were gated based on their positivity for CD56 and negativity for CD3 (C).

Activation markers (CD25 - D, NKp44 - E, NKG2D – F, all represent by grey population) were evaluated on final NK cells where isotype controls (white peak with a black line) or unstained controls were used more precise gating strategy.

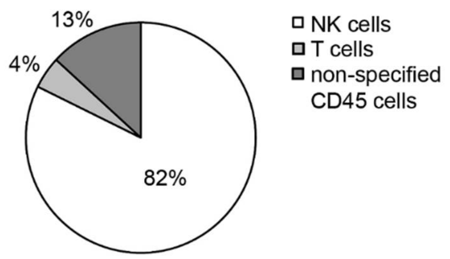

Supplementary figure S2. Cellular composition after ten days of NK cells cultured in the presence of IL-2 and pooled feeder cells. Median of 8 donors. The purity was in the range of 68-92%.

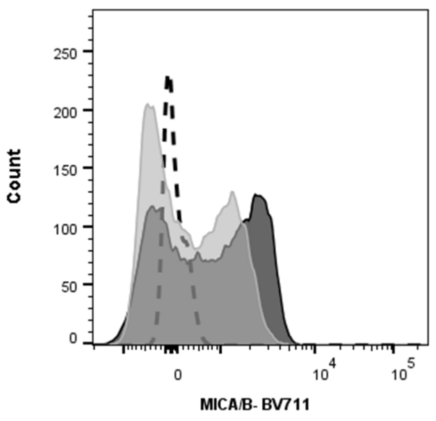

Supplementary figure S3. Expression of MICA/B on the surface of KG1a cells without (light grey) or after treatment with Ara-C (0.5µM; dark grey). Very low differences in fluorescence intensity were observed (treated cells MFI=1796, untreated cells MFI=1321) but a number of positive cells was higher in treated cells compared with untreated control. Isotype control was used for more precise gating (white peak with a dashed black line).

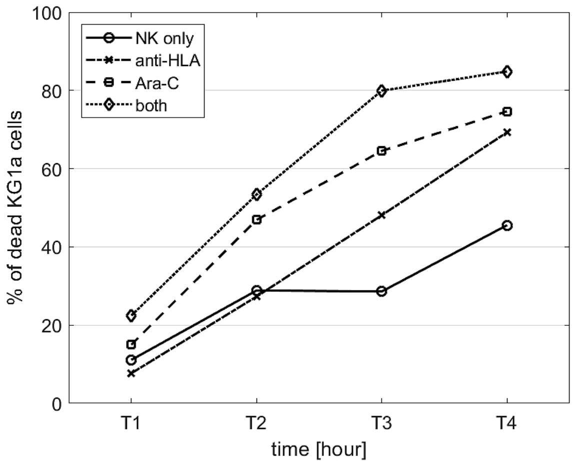

Supplementary figure S4. Time dependence evolution of dead KG1a cells under different culture condition.

The number of dead cells increased in a time-dependent manner in all culture condition. The highest difference between control and treated cells was observed in the last time-point, where the number of dead cells in combined therapies reached to 85%. n=8
